# Complementing the phenotypical spectrum of *TUBA1A* tubulinopathy and its role in early-onset epilepsies

**DOI:** 10.1038/s41431-021-01027-0

**Published:** 2022-01-11

**Authors:** Julian Schröter, Bernt Popp, Heiko Brennenstuhl, Jan H. Döring, Stephany H. Donze, Emilia K. Bijlsma, Arie van Haeringen, Dagmar Huhle, Leonie Jestaedt, Andreas Merkenschlager, Maria Arelin, Daniel Gräfe, Sonja Neuser, Stephanie Oates, Deb K. Pal, Michael J. Parker, Johannes R. Lemke, Georg F. Hoffmann, Stefan Kölker, Inga Harting, Steffen Syrbe

**Affiliations:** 1 Division of Pediatric Epileptology, Center for Pediatrics and Adolescent Medicine, University Hospital Heidelberg, Heidelberg, Germany; 2 Institute of Human Genetics, University of Leipzig Medical Center, Leipzig, Germany; 3 Division of Neuropediatrics and Inherited Metabolic Diseases, Center for Pediatrics and Adolescent Medicine, University Hospital Heidelberg, Heidelberg, Germany; 4 Department of Clinical Genetics, Leiden University Medical Center, Leiden, The Netherlands; 5 Medizinisches Versorgungszentrum, Karl-Liebknecht-Str. 14, 04107 Leipzig, Germany; 6 Department of Neuroradiology, University Hospital Heidelberg, Heidelberg, Germany; 7 Department of Women and Child Health, Hospital for Children and Adolescents, University Hospital Leipzig, Leipzig, Germany; 8 Department of Pediatric Radiology, Hospital for Children and Adolescents, University Hospital Leipzig, Leipzig, Germany; 9 King’s College Hospital, London, UK; 10 Evelina London Children’s Hospital, London, UK; 11 Department of Basic & Clinical Neurosciences, Institute of Psychiatry, Psychology & Neuroscience, King’s College London, London, UK; 12 MRC Centre for Neurodevelopmental Disorders, King’s College London, London, UK; 13 Sheffield Children’s Hospital NHS Foundation Trust, Western Bank, Sheffield, UK; 14 Center for Rare Diseases, University of Leipzig Medical Center, Leipzig, Germany

**Keywords:** Neurodevelopmental disorders, Epilepsy, Genetics research

## Abstract

*TUBA1A* tubulinopathy is a rare neurodevelopmental disorder associated with brain malformations as well as early-onset and intractable epilepsy. As pathomechanisms and genotype-phenotype correlations are not completely understood, we aimed to provide further insights into the phenotypic and genetic spectrum. We here present a multicenter case series of ten unrelated individuals from four European countries using systematic MRI re-evaluation, protein structure analysis, and prediction score modeling. In two cases, pregnancy was terminated due to brain malformations. Amongst the eight living individuals, the phenotypic range showed various severity. Global developmental delay and severe motor impairment with tetraparesis was present in 63% and 50% of the subjects, respectively. Epilepsy was observed in 75% of the cases, which showed infantile onset in 83% and a refractory course in 50%. One individual presented a novel *TUBA1A*-associated electroclinical phenotype with evolvement from early myoclonic encephalopathy to continuous spike-and-wave during sleep. Neuroradiological features comprised a heterogeneous spectrum of cortical and extracortical malformations including rare findings such as cobblestone lissencephaly and subcortical band heterotopia. Two individuals developed hydrocephalus with subsequent posterior infarction. We report four novel and five previously published *TUBA1A* missense variants whose resulting amino acid substitutions likely affect longitudinal, lateral, and motor protein interactions as well as GTP binding. Assessment of pathogenic and benign variant distributions in synopsis with prediction scores revealed sections of variant enrichment and intolerance to missense variation. We here extend the clinical, neuroradiological, and genetic spectrum of *TUBA1A* tubulinopathy and provide insights into residue-specific pathomechanisms and genotype-phenotype correlations.

## Introduction

Microtubules play a pivotal role during brain development being indispensable for mitosis, neuronal migration, synaptic connectivity, and axonal transport [1]. Perturbance of microtubule-dependent functions from variants in the main components α-, β-, and γ-tubulin cause neurodevelopmental disorders (NDDs) with partially overlapping phenotypes, known as tubulinopathies [2–4]. Tubulinopathies show a broad spectrum of malformations of cortical (MCDs) and extra-cortical development and are increasingly recognized as a cause of early-onset epilepsies [5, 6]. Their anti-epileptic treatment is challenging as epilepsy predominantly shows an infantile onset and treatment-resistant course with various semiologies [7]. As sole specific epilepsy syndrome, infantile spasms were reported in one third of the cases [8]. The different tubulin isotypes show sequence homology but differ in spatio-temporal expression suggesting unique, isotype-specific functions [9]. Tubulinopathies are predominantly caused by variants in *TUBA1A*, encoding the major CNS α-tubulin isotype, which accounts for a rapidly growing number of more than 170 cases [8]. *TUBA1A* tubulinopathy shows a more severe clinical and neuroradiological phenotype than β-tubulinopathies comprising a combination of MCDs and changes of extra-cortical structures such as cerebellum, corpus callosum (CC), basal ganglia, brainstem, and ventricles. MCDs encompass subtypes of lissencephaly and cortical irregularities with simplified gyration in combination with atypical polymicrogyria [5]. For this so called “polymicrogyria-like cortical dysplasia”, the term “tubulinopathy-associated cortical dysgenesis” has been proposed [10]. Except for three familial cases, *TUBA1A*-tubulinopathy is exclusively caused by de novo missense variants scattered throughout the gene [11]. So far, only one case with the variant p.(Tyr408*), resulting in a truncated protein, has been reported with polymicrogyria, epilepsy, and global developmental delay [12]. Given the absence of truncating variants in affected and healthy individuals, haploinsufficiency is likely not tolerated in humans. Establishment of genotype-phenotype correlations is challenging and underlying pathomechanisms of variant-specific effects are not completely understood [11]. Depending on their localization in the quaternary structure, variants are predicted to alter longitudinal, lateral, or microtubule surface interactions, thereby disturbing interaction with different motor and microtubule-associated proteins (MAPs) potentiating phenotypic diversity [13].

In this study, we complement clinical and genetic characteristics of *TUBA1A* tubulinopathy in a series of ten individuals with nine different missense variants. By deep-phenotyping neuroradiological and electroclinical findings, we further elucidated the spectrum of this disorder associated with early-onset epilepsies.

## Subjects and methods

### Recruitment and genetic testing

Individuals were recruited from collaborating centers in Germany, Switzerland, the Netherlands, and the United Kingdom. Clinical data and imaging results were obtained from medical records. Seizure outcome relates to the last 6 months of follow-up and was defined as follows: “controlled” = no/sporadic seizures, “partially controlled” ≥ 50% seizure reduction, “refractory to treatment” ≤ 50% seizure reduction. Genetic testing was performed using either gene panel, single, or trio-exome sequencing of peripheral blood samples and amniotic fluids prepared by standard techniques. Putatively causative variants were confirmed by Sanger sequencing except for i01 and i08. Segregation was tested in all but i05. Variants were classified based on the ACMG criteria [14]. Genetic and phenotypic information of all variants in this report have been submitted to the ClinVar database. All participating families gave their informed consent for genetic testing and use of pseudonymized clinical data.

For specific missense variants, protein structure and variant effect prediction modeling as well as literature research was performed. Methodological details are provided in the Supplementary Methods.

### Systematic, quantitative assessment of neuroradiological features

In addition to standard assessment of MRI in all cases, nine postnatal MRIs of the four individuals i02, i03, i05, and i06 (age at imaging: 4 days-4.8 years; median: 72.9 months) and one fetal MRI (i09) were systematically assessed by two experienced neuroradiologists (LJ, IH). MRIs were reviewed for abnormalities of (1.) cerebral cortex including hippocampus, (2.) deep gray matter (thalamus and basal ganglia), (3.) internal capsule, (4.) brainstem, (5.) posterior fossa, (6.) cerebellum, (7.) white matter, (8.) cranial nerves, and (9.) internal and external CSF spaces. Further methodological details regarding descriptive and quantitative MRI re-evaluation parameters are delineated in the Supplementary Methods.

## Results

The cohort comprised ten individuals from which eight were alive at last follow-up (age range: 1–14 yrs; median: 9.5 yrs). In two cases (i08, i09), pregnancy was terminated in GW 19 + 2 and 25 + 0, respectively, due to severe brain malformations detected in prenatal ultrasound and fetal MRI. Only the eight living individuals had postnatal clinical examinations. Table 1 summarizes clinical, neuroradiological, and genetic characteristics.

**Table 1:** Genetic, clinical, and neuroradiological summary.

Individual	i01	i02	i03	i04	i05	i06	i07	i08 (fetus)	i09 (fetus)	i10
Variant	c.5 G > A p.(Arg2His)	c.518 C > T p.(Pro173Leu)	c.521 C > T p.(Ala174Val)	c.521 C > T p.(Ala174Val)	c.652 G > A p.(Asp218Asn)	c.656 T > C p.(Ile219Thr)	c.967 G > A p.(Val323Met)	c.1049 G > T p.(Gly350Val)	c.1205 G > A p.(Arg402His)	c.1264 C > A p.(Arg422Ser)
Sex	M	F	F	M	M	F	M	F	n/a	M
Inheritance	De novo	De novo	De novo	De novo	n/a	De novo	De novo	De novo	De novo	De novo
Age (last FU)	9 yrs	11 yrs	3 yrs	12 mo	11 yrs	14 yrs	10 yrs	GW 19 + 2	GW 25 + 0	6 yrs
Microcephaly	Yes	Yes	Yes	Yes	No	Yes	Yes	No	n/a	Yes
Head size [SDS]	n/a	−2.84	−5.83	−6.60	−0.23	−4.56	−2.50	n/a		−2.97
Epilepsy	Yes	No	Yes	Yes	Yes	Yes	No	n/a	n/a	Yes
Developmental delay	Yes	Yes	Yes	n/a	Yes	Yes	Yes	n/a	n/a	Yesl
Affected area(s)	Speech	Global	Global		Global	Global	Isolated ID			Global
Behavioral disorder	ASD	No	No	n/a	No	No	ASD	n/a	n/a	No
Abnormal muscular tone	Hypotonia	Hypotonia	Hypo-/Hypertonia Spastic-dyskin. tetraparesis	Opisthotonus	Spastic-hypotonic tetraparesis	Hypotonia Dystonic tetraparesis	No	n/a	n/a	Spastic tetraparesis
Motor impairment	Yes (Ataxia)	Yes	Yes	Yes	Yes	Yes	Yes (Leg dyspraxia)	n/a	n/a	Yes
Ambulatory?	Yes	Yes	No	n/a	No	No	Yes	n/a	n/a	No
Ocular motility disorder	n/a	n/a	Strabismus	Nystagmus	Nystagmus	Nystagmus	Strabismus	n/a	n/a	n/a
Vision	n/a	n/a	Central vision disorder	Normal	Central vision disorder	Central vision disorder	Normal	n/a	n/a	n/a
Dysmorphisms	n/a	n/a	Yes	Yes	No	Yes	Yes	Yes	n/a	No
Brain imaging										
Age at last imaging	4.8 yrs	4.8 yrs	5 mo	3 d	11.7 yrs	14.8 yrs	13 mo	GW 19 + 2 (US)	GW 25 + 0	21 mo
Cerebral cortex	Focal cortical dysplasia	PMG (perisylvian)	Simplified GP PMG (perisylvian)	SBH Pachygyria (frontal)	Simplified GP Cobblestone-LIS	Simplified GP PMG (perisylvian)	Normal	Normal	Simplified GP	Dysgyria (left temporal lobe)
Hippocampus	n/a	Normal	Hypoplastic	n/a	Hypoplastic	Hypoplastic	n/a	n/a	n/a	Dysmorphic
Deep gray matter	Dysmorph. BG	Normal	Small thalami Dysmorph. BG	n/a	Small thalami Dysmorph. BG	Small thalami Dysmorph. BG	n/a	n/a	Normal	Dysmorph. BG
Brainstem	Normal	Normal	Hypoplastic	n/a	Hypoplastic, asymmetric	Hypoplastic, asymmetric	n/a	n/a	Thick tectum	Normal
Vermis	Hypoplastic, rotated	Normal	Hypoplastic, rotated	Hypoplastic	Hypoplastic, rotated	Hypoplastic, rotated	n/a	n/a	Hypoplastic	Normal
Cerebellar hemispheres	Abnormal foliation	Normal	Abnormal foliation	Hypoplastic	Abnormal foliation	Abnormal foliation	n/a	n/a	n/a	Normal
Corpus callosum	Hypoplastic	Normal	Hypoplastic	n/a	Hypoplastic	Agenetic	Hypoplastic	n/a	Hypogenetic	Normal
Myelination	n/a	Complete	Delayed	Delayed	Complete	Complete		n/a	n/a	Complete
Ventricles	Normal	Normal	Large (II, III, IV)	n/a	Large (II, III, IV)	Large (II, IV)	Large (I, II)	Large (III)	Large (II)	Normal
External CSF spaces	n/a	Normal	Large	n/a	Normal	Large	n/a	Cysts	Normal	n/a

*ASD* autism spectrum disorder, *BG* basal ganglia, *CSF* cerebral spinal fluid, *dyskin.* dyskinetic, *dysmorph.* dysmorphic, *F* female, *FU* follow-up, *GP* gyral pattern, *ID* intellectual disability, *LIS* lissencephaly, *M* male, *n/a* not assessed, *SBH* subcortical band heterotopia, *SDS* standard deviation score, *TOP* termination of pregnancy, *US* ultrasound.

### Clinical features

Seven out of eight living individuals (88%) showed microcephaly with an occipito-frontal circumference range from −6.60 to −2.50 SDS at last follow-up (mean: −4.22 SDS). All individuals had developmental disorders affecting cognition, which comprised global developmental delay in 5/8 (63%) and autism spectrum disorder in 2/8 individuals (25%). Motor symptoms were ubiquitously observed ranging from isolated muscular hypotonia (i01, i02) or leg dyspraxia (i07) in the three ambulatory subjects, older than 12 months, to tetraparesis with spastic, dystonic, spastic-dyskinetic or spastic-hypotonic characteristics in the four non-ambulatory subjects. Independent walking was achieved in all ambulatory subjects.

Epilepsy was present in 6/8 subjects (75%). Age at onset was known in 5/6 cases that all had infantile and even neonatal seizures (3/5; i03, i05, and i04) with a median age of 7 days (range: 3 d–8 mo). Four subjects had combined focal and generalized epilepsies with multiple seizure types including focal to bilateral tonic-clonic seizures (three cases) and epileptic spasms (two cases). Developmental and epileptic encephalopathy was reported in all six individuals with distinct early-onset epileptic encephalopathy in two children classified as West syndrome (i06) and early myoclonic encephalopathy with burst suppression (i03). The electroclinical pattern of subject i03 subsequently evolved to encephalopathy with continuous spike-and-wave during sleep (CSWS) during childhood (Fig. S3). More than 50% reduction in seizure frequency was achieved in 2/5 cases (40%) with monotherapy using valproic acid or vigabatrin whereas seizures were refractory in the remaining cases despite polypharmacy with two or more anti-epileptic drugs. Further epileptological features are presented in Table 2.

**Table 2:** Epileptological features of living individuals.

Individual^a^	i01	i03	i04	i05	i06	i10
Variant	c.5 G > A p.(Arg2His)	c.521 C > T p.(Ala174Val)	c.521 C > T p.(Ala174Val)	c.652 G > A p.(Asp218Asn)	c.656 T > C p.(Ile219Thr)	c.1264 C > A p.(Arg422Ser)
Epilepsy	Yes	Yes	Yes	Yes	Yes	Yes
Age at seizure onset	8 mo	5 d	3 d	7 d	2 mo	n/a
Seizure types	1. Focal to bilateral tonic-clonic szs. 2. Focal autonomic szs.	1. Myoclonic szs. (neonatal) 2. Focal tonic szs. w/ impaired awareness 3. Epileptic spasms	Generalized tonic-clonic szs.	1. Focal to bilateral tonic-clonic szs. 2. Focal myoclonic szs.	1. Epileptic spasms 2. Focal to bilateral tonic-clonic and myoclonic szs.	Focal szs. w/ impaired awareness
Epilepsy type	Combined focal and generalized epilepsy	Combined focal and generalized epilepsy	Generalized epilepsy	Combined focal and generalized epilepsy	Combined focal and generalized epilepsy	Focal epilepsy
Primary	Unspecified DEE	Early myoclonic encephalopathy w/ burst suppression	Unspecified DEE	Unspecified DEE	West syndrome	Unspecified DE + E
Epilepsy syndrome						
Epilepsy outcome	Refractory to treatment	Partially controlled	n/a	Refractory to treatment	Refractory to treatment	Partially controlled
Development prior to seizure onset	Delayed	Neonatal onset	Neonatal onset	Neonatal onset	Unremarkable	Delayed
EEG at onset	Left sided temporal slowing with frequent high voltage spikes and slow-waves	Constant suppression pattern, evolving to burst-suppression pattern in infancy	n/a	n/a	Hypsarrhythmia, multifocal spikes, generalized slowing	n/a
EEG at follow-up	Right sided posterior slow-waves and occipital paroxysms of spikes	Multifocal, bihemispheric spikes, CSWS (age > 3 yrs)	n/a	Dissociated brain activity, bilateral hypsarrhythmia pattern during sleep, suppression-burst pattern (left hemisphere)	Generalized slowing, occasional multifocal spikes, atypical hypsarrhythmia	n/a
Current AED	Vigabatrin, levetiracetam, valproic acid, lamotrigine	Vigabatrin	Topiramate, phenobarbital	Lamotrigine, levetiracetam, clobazam, cannabidiol	Topiramate, levetiracetam	Valproic acid
AED synopsis	n/a	Levetiracetam, methylprednisolone	n/a	Valproic acid, vigabatrin, topiramate, lacosamide, phenobarbital, sultiame	Vigabatrin, phenobarbital, valproic acid, sultiame	n/a

*AED* anti-epileptic drug, *DEE* developmental and epileptic encephalopathy, *DE* *+* *E* developmental encephalopathy with epilepsy, *CSWS* continuous spike-and-wave during sleep, *FU* follow-up, *n/a* not assessed, *w/* with.

^a^Four individuals were excluded from this synopsis due to termination of pregnancy (i08, i09) and absence of epilepsy (i02, i07), respectively.

Ocular abnormalities were frequent (5/8; 63%) and included ocular motility disorders such as nystagmus (3/5; 60%) and strabismus (2/5; 40%) as well as central visual impairment (3/5; 60%) due to optic nerve hypoplasia (i03, i06) or retinal atrophy (i05). Dysmorphic features included facial dysmorphisms such as low-set ears, small mouth, high palate (i06), flat profile of the face (i08), bitemporal narrowing, pronounced upper orbital rim, upslanting epicanthal fold, macrotia, and retrognathia (i04). Further dysmorphisms comprised conic fingers, sacral pit (i04), spina bifida occulta (i07), brachycephalus, overriding fingers, and club feet (i08).

### Neuroradiological features

**Fig. 1:**
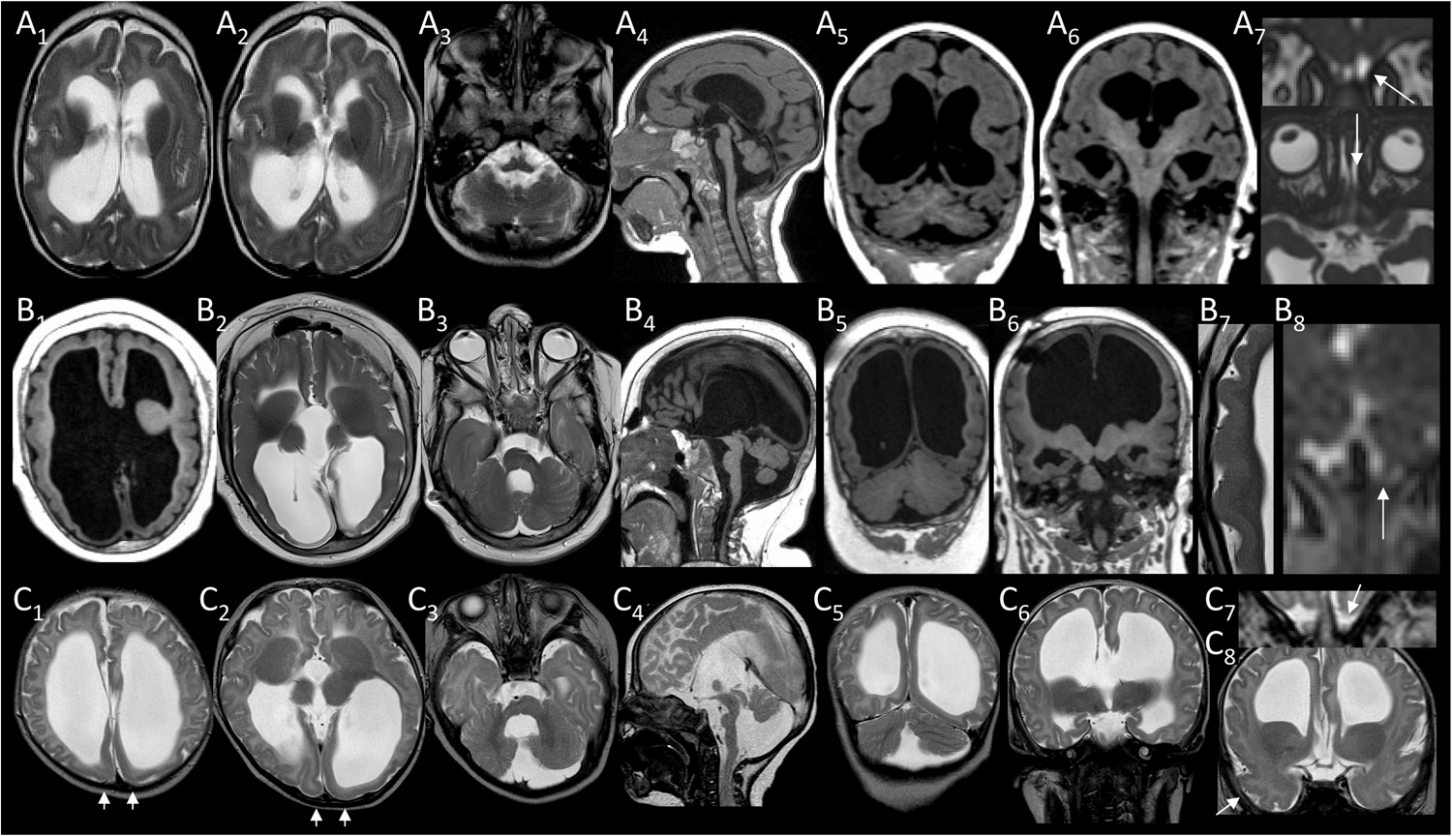
Brain imaging of individuals with *TUBA1A* variants. Individuals i03 (**A**), i05 (**B**), and i06 (**C**) have common findings of (I) small thalami, (II) dysplastic, namely rounded, rotated basal ganglia without discernible ALIC and altered shape of frontal horns, (III) abnormal brainstem, (IV) a small, rotated vermis, as well as (V) malformations of cortical development. **A** *i03 at 5 months: *Simplified gyral pattern and thick cortex with irregular surface and cortex-white matter junction in the perisylvian area, suggestive of polymicrogyria (A_1,2,5_). Small thalami, rounded basal ganglia without discernible ALIC (A_2_). CC and brainstem are thin, whereas mesencephalon and medulla oblongata are disproportionately thick and long, respectively (A_4_). Cerebellar foliation is irregular, with a polymicrogyria-like aspect (A_3,5_). The hippocampus is hypoplastic with incomplete enfolding and near vertical orientation (A_6_). The left olfactory bulb is missing (A_7_; arrows). **B** *i05 at 9 years:* Shunted hydrocephalus due to aqueduct stenosis (shunt not depicted) with thick skull and T2-hyperintense residua of bilateral posterior cerebral artery infarction (B_1–3_). Gyral pattern is simplified, the cortex is thick with slightly irregular inner surface and faint radial stripes suggestive of cobblestone lissencephaly (B_7_). Small thalami and rounded, rotated basal ganglia without discernible ALIC (B_2_). Thin, distended CC, presumably a combination of hypoplasia and hydrocephalus (B_4_). Abnormal brainstem with short pons as well as disproportionately thick mesencephalon and long medulla oblongata (B_4_); asymmetry is best appreciated on axial image (B_3_). Cerebellar folia are irregular (B_3,5_). The hippocampus is hypoplastic with incomplete enfolding and near vertical orientation (B_6_). Both olfactory bulbs are visible (B_8_; arrow). **C** *i06 at 2.8 months* before shunting of hydrocephalus (motion artefacts on follow-up): Decreased sulci with thick, smooth cortex of the parieto-occipital lobes (C_1,2_; arrows) suggestive of pachygyria and irregular internal and external cortical surface in right temporal lobe and bilateral perisylvian areas suggestive of polymicrogyria (C_2,8_; arrows). Agenesis of the CC (C_4,6_). Abnormal brainstem with disproportionately thick mesencephalon, short pons, long medulla oblongata (C_4_), and asymmetry (C_3_). Hypoplastic hippocampus and parahippocampal gyrus (C_6_) and hypoplastic left olfactory bulb (C_7,8_; arrow). *ALIC* = *anterior limb of the internal capsule, CC* = *corpus callosum*.

All postnatally examined individuals had MCDs varying from focal, mainly perisylvian, irregular sulci and/or cortex-white matter junctions suggestive of polymicrogyria (i02, i03, i06) to a diffusely simplified gyral pattern with variable thickening and irregularity of the cortex (i03, i05, i06; Figs. 1 & S1). A thick, lissencephalic cortex with an irregular inner surface and faint radial stripes consistent with cobblestone lissencephaly was observed in i05. In i03, i05, and i06, the hippocampus was hypoplastic (Fig. 1). T2-hyperintensity and facilitated diffusion in the territory of the posterior cerebral arteries were observed in i05 and i06 showing hydrocephalus (Fig. S1). All postnatal MRIs displayed abnormal deep gray matter: Changes were subtle in i02 where the caudal portion of the anterior limb of the internal capsule (ALIC) was not discernible (Fig. S2). i03, i05, and i06 had small thalami and dysplastic, relatively larger, round and/or rotated basal ganglia without discernible ALIC and with dysplastic anterior horns of the lateral ventricles (Fig. 1). The brainstem was thin (*z*-scores ≤ −2.0 SDS) in all except for i09 and asymmetric in i05 and i06, while the mesencephalon was overall better preserved (Fig. 1, Table S1). Abnormal foliation of the craniomedial cerebellum and a small, rotated vermis were present in the three severely affected individuals i03, i05, and i06, the latter with large posterior fossa (Fig. 1). The CC was either normal (i02), thin (i03, i05, i09), or agenetic (i06; Fig. 1 & S2). Myelination was delayed in i03 and i06 but complete in all individuals after the age of 2 years. The left olfactory bulb was absent in i03 and thin in i06 (Fig. 1). In i05 and i06, hydrocephalus developed from aqueduct stenosis, requiring ventriculo-peritoneal shunting (Fig. S1). Fetal imaging of i09 revealed a not age-appropriate gyral pattern, hypoplasia of the CC, wide occipital horns of the lateral ventricles and a small vermis. Thalami and basal ganglia showed no discernible abnormalities (Table S1). Detailed features from MRI re-evaluation are further delineated in Figs. 1, S1, S2, and Table S1.

Neuroradiological findings of the five individuals not systematically re-assessed was performed between the age of 3 days and 4.8 years. All MRIs were abnormal. MCDs were described in 3/5 cases (60%) including focal cortical dysplasia (i01), focal dysgyria with dysmorphic hippocampus (i10), and frontal pachygyria with subcortical band heterotopia (i04). Basal ganglia were dysmorphic in two cases with asymmetry (i10) and indiscernible ALIC (i01). Abnormalities of the brainstem were not described. Cerebellar malformations were present in 3/4 cases (75%) and ranged from hypoplasia (i04) to irregular foliation with hypoplastic and rotated vermis (i01). The CC was hypoplastic in i01 and i07 and normal in i10. Myelination was delayed in i04. Dilated ventricles were observed in i07 and i08.

### Genetics

**Fig. 2:**
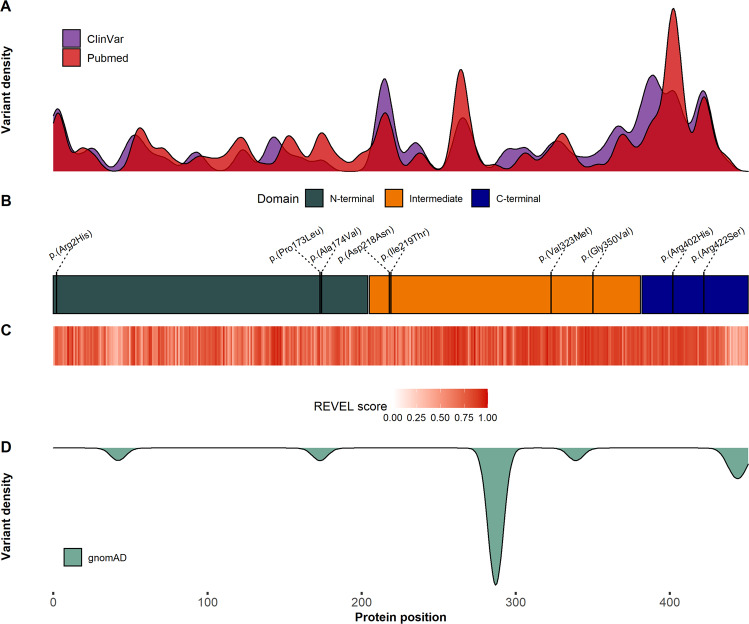
Distribution patterns of pathogenic and benign *TUBA1A* variants. **A** Density of pathogenic variants reported in Pubmed (red) and ClinVar (violet). **B** Linearized TUBA1A protein model including domain annotation and description of herein reported variants (black). **C** Heatmap visualization of mean REVEL score values for all biologically possible *TUBA1A* missense variants according to their position in the primary structure. **D** Density of benign variants reported in gnomAD (light green). Pathogenic variant enrichment can be found in the C-terminal domain and, in particular, at residues Arg2, Arg214, Arg264, Arg402, and Arg422 (*N* > 10). Distribution of pathogenic compared to benign variants is mainly reciprocal. Variant densities are plotted with respect to their allele count.

**Fig. 3:**
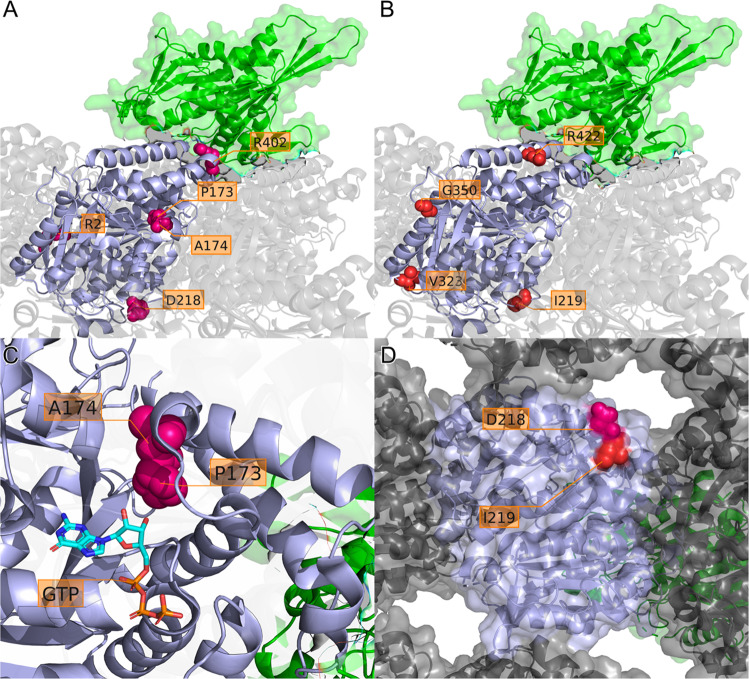
Localization of missense variants in a TUBA1A protein model. Ribbon diagram of the TUBA1A monomer (highlighted in light blue) surrounded by α- and β-tubulin monomers assembling the microtubule filament (transparent gray; based on PDB: 5JCO). The kinesin KIF1A motor protein is aligned to this structure and shown as ribbon with transparent surface (green; PDB: 2HXF). Amino acid residues affected by missense variants are shown as spheres in (**A**) for variants previously reported in the literature (magenta) and (**B**) for novel variants reported here (red). **C** View from A/B rotated by −90° *x*-axis and −45° *y*-axis with the amino acid positions A174 and P173 as spheres and the GTP molecule in stick representation. Both residues are proximal to the GTP binding site and affect the same T5 turn between two α-helix folds. The amino acid changes at these positions likely disrupt binding to the GTP ribose group. **D** View from A/B rotated by −90° *x*-axis showing the central TUBA1A monomer (with transparent surface) from luminal with the two amino acid positions D218 and I219 predicted to disrupt the longitudinal interactions at the surface close to the neighboring monomer.

Nine different pathogenic or likely pathogenic missense variants in *TUBA1A* were detected in ten unrelated individuals. While the four variants c.656 T > C p.(Ile219Thr), c.967 G > A p.(Val323Met), c.1049 G > T p.(Gly350Val), and c.1264 C > A p.(Arg422Ser) were novel, the five variants c.5 G > A p.(Arg2His), c.518 C > T p.(Pro173Leu), c.521 C > T p.(Ala174Val), c.652 G > A p.(Asp218Asn), and c.1205 G > A p.(Arg402His) were previously reported [2, 15–18]. The variant p.(Ala174Val) occurred twice in our cohort (i03, i04; Table S2). All variants that were tested for segregation occurred *de novo*. Affected residues are highly conserved throughout species and isotypes [9]. We visualized the distribution of all 93 pathogenic and 65 likely pathogenic variants that have been reported in the literature and ClinVar; while pathogenic variants affect large parts of the protein, seven different variants from healthy gnomAD controls show a reciprocal distribution and especially enrich at residue Ser287 (*N* = 11) and in the variable C-terminal tail including residues 440-448 (Fig. 2, Table S4). VEP score values of gnomAD variants estimate a mild or moderate damaging effect in comparison to all remaining substitutions at the respective residue. As an exception, p.(Pro173Ser) is listed once in gnomAD although a high damaging effect is predicted and different missense variants at this residue have repeatedly been associated with NDDs. Pathogenic variants emphatically gather around residues Arg2, Arg214, Arg264, Arg390, Arg402, and Arg422 (all *N* > 10; Fig. 2). Although the allele frequency of pathogenic variants is similarly distributed throughout the N-terminal (35%), intermediate (33%), and C-terminal domain (32%), the highest mutational constraint is observed in the shorter C-terminal domain containing 16% of amino acids. To evaluate the likelihood of pathogenic variant effects, we annotated the VEP meta-score REVEL for all theoretically possible *TUBA1A* missense variants and assessed position-specific score value distributions using a heatmap. The variants identified in this study are localized in sections of moderate to high predicted damaging effects and high REVEL score values overlap with sections of pathogenic variant enrichment with C-terminal emphasis, which is supported by similar tendencies of different established VEP scores (Fig. 2; Fig. S4, Tables S2 & S3). All variants in our cohort were assessed for localization and effects on the TUBA1A quaternary structure using protein modeling (Fig. 3). The known variants are distributed throughout TUBA1A belonging to different mutational effect classes. p.(Arg402His) and p.(Arg422Ser) are located in the C-terminal H11-H11’ loop and helix H12, respectively, putatively interacting with motor proteins and MAPs. The novel variants p.(Val323Met) and p.(Gly350Val) are located in β-strands S8 and S9, respectively, mediating conformational changes upon GTP hydrolysis and stabilizing lateral interactions. p.(Pro173Leu) and p.(Ala174Val) show proximity to a GTP binding site interacting with the GTP ribose group. p.(Asp218Asn) and p.(Ile219Thr) are located at the luminal side of the longitudinal interface known to mediate intradimer interactions between α- and β-tubulin monomers whereas p.(Arg2His) is located in an area essential for longitudinal interdimer interactions.

## Discussion

*TUBA1A* tubulinopathy is a rare neurodevelopmental disorder with a high burden of disease but clinical studies with sufficient cohort sizes are scarce. We here complement the clinical, epileptological, neuroradiological, and genetic spectrum presenting ten individuals with four novel and five previously published missense variants.

The broad range of phenotypic severity observed here underscores the clinical variability of *TUBA1A* tubulinopathy [5, 8]. ASD in association with isolated hypoplasia of the CC, as observed in i07, represents the mild end of the spectrum emphasizing the relevance of *TUBA1A* in ASD pathophysiology [19]. The severe spectrum comprises extensive cortical dysgyria and hydrocephalus in association with severe global developmental delay, tetraparesis, and intractable epilepsy (i05, i06). More extensive MCDs are a relevant cause for termination of pregnancy, mirrored by the two fetal cases described here. *TUBA1A*-associated epilepsy shows various semiologies and predominantly manifests in the first year of life, which is supported by the results in our study [7, 8]. We underline the role of tubulinopathies in infantile epilepsy reporting a high proportion of children with neonatal onset and refractory course of epilepsy. Notably, i03 showed early myoclonic encephalopathy evolving to an electroclinical picture of CSWS during childhood with a high index of epileptiform potentials during sleep. CSWS has not been described in *TUBA1A* tubulinopathy so far but has been noted as a feature of other monogenic NDDs such as *MECP2*-associated Rett syndrome or Angelman syndrome [7, 20]. Brain malformations were highly variable ranging from focal to extensive brain malformations. MCDs reported here were characterized by an irregular pattern of gyri and sulci different from classic polymicrogyria or lissencephaly, as also suggested by Oegema et al. [10]. Interestingly, i05 and i06 had hydrocephalus from connatal aqueduct stenosis that became symptomatic during the first weeks of life. Another distinct feature that has not previously been described in *TUBA1A* tubulinopathy was ischemia in the territory of the posterior cerebral arteries in both cases [21]. The risk for hydrocephalus and associated complications should therefore be acknowledged by caring physicians. Subcortical band heterotopia has only been sporadically described in tubulinopathies, being more frequent in association with the genes *DCX* and *LIS1* [22]. By presenting another case (i04), we here identify *TUBA1A* tubulinopathy as a rare but recurrent cause for this rare migrational disorder [23]. Tubulins have a crucial role in cerebellar development and circuit formation that has been demonstrated by fetal neuropathology, RNA sequencing, and mouse models [18, 24, 25]. Consistently, cerebellar involvement was an initial, non-progressive finding in the majority of individuals reported here [26].

Due to its high phylogenetic conservation, *TUBA1A* has little tolerance for missense variation. Accordingly, the general population is depleted of missense variants mirrored by only seven gnomAD entries. Consequently, interpretation of novel variants is challenging, also because VEP scores generally predict a pronounced deleterious effect. By outlining distributions of pathogenic and benign variants in synopsis with VEP scores for all possible missense variants, we show that both features in combination, despite relevant limitations, can be useful for variant interpretation. Our heatmap visualization models approximately recapitulate regions with pathogenic variant enrichment. The residues Arg264, Arg402, and Arg422 are most frequently affected by missense variants, accounting for 55% (*N* = 57) of all cases reported in the literature and ClinVar. Important interactions of these residues with motor proteins, MAPs, and chaperonins have been demonstrated by protein structure and animal models explaining their role in pathophysiology of tubulinopathies [5, 27, 28] This is in line with the recurrent variant p.(Arg402His) identified in a fetus with severe brain malformations and the novel variant p.(Arg422Ser) found in a living individual of our cohort. The novel variants p.(Val323Met) and p.(Gly350Val) are located in TUBA1A sections undergoing conformational change upon GTP hydrolysis [29]. As additional structural feature, Gly350 stabilizes the M-loop being essential for lateral interactions between microtubule protofilaments [30]. Regarding the predicted damaging effect of p.(Gly350Val) and the severe brain malformations of the fetus, dysfunction of lateral interactions and an impaired conformational change might be probable pathomechanisms. In contrast, the substitutions p.(Val353Ile) and p.(Val323Met) were associated with milder NDDs and lower VEP score values [5]. Besides motor protein and MAP interactions, longitudinal intra- and interdimer interactions of α-β-heterodimers are postulated to be crucial for microtubule lattice stability [29]. Remarkably, i05 and i06 carrying substitutions at residues Asp218 and Ile219, involved in longitudinal interactions, both show a severe, overlapping phenotype and course of disease [31]. Furthermore, an individual with the alternate substitution p.(Asp218Tyr) had a similar phenotype to i05 including a thin cortex layer and dysgyria resembling cobblestone lissencephaly [32]. Despite reports on two milder affected individuals, our findings concerning Asp218 and Ile219 support relevance of longitudinal interactions for proper TUBA1A function [18, 33]. We present three individuals harboring the known and recently reported substitutions p.(Pro173Leu) and p.(Ala174Val) at residues likely involved in GTP binding [29]. To date, only few substitutions of this functional class have been reported [13]. i03 and i04, both carrying p.(Ala174Val), are severely affected and i03 shares high phenotypic overlap with the published case [17]. i02 harboring p.(Pro173Leu) supports genotype-phenotype correlations comprising rather mild brain malformations with moderate developmental delay and ASD, respectively, comparable to previously reported individuals carrying this variant [16, 19, 34], from which only one showed a severe phenotype [11]. High phenotypic variability from alterations at this residue is mirrored by high VEP scores for the substitution with Leucine in contrast to p.(Pro173Ser) found in a single gnomAD control.

## Conclusion

*TUBA1A* tubulinopathy is a relevant cause of congenital brain malformations as well as early-onset and intractable epilepsy with semiologic diversity. We here assess a representative cohort and highlight novel epileptological, neuroradiological, and genetic aspects. As establishment of genotype-phenotype correlations remains challenging, we aimed to facilitate future interpretation of novel variants using distributional and computational prediction analysis in affected individuals and controls. Missense variants in additional genes, random processes in cellular pathways, epigenetic factors, and chaperonin interactions could be possible modifiers and will require further research to completely understand the role of *TUBA1A* variation in neurological diseases [13, 35, 36].

## Supplementary information

Supplementary Information

Supplementary Table S1

Supplementary Table S2

Supplementary Table S3

Supplementary Table S4

## Data Availability

All variants identified in this report have been submitted to the ClinVar database including ACMG assertation and summarized clinical data. All further data necessary for interpretation are provided within paper and supplementary information including supplementary tables. Additional data are available from the corresponding author on reasonable request.
