# Supplementary material for: Complementing the phenotypical spectrum of TUBA1A tubulinopathy and its role in early-onset epilepsies
Source: Eur J Hum Genet. 2022 Jan 11;30(3):298–306. doi: 10.1038/s41431-021-01027-0 (PMC8904761; doi:10.1038/s41431-021-01027-0)
Supplement: Supplementary file 1 — Supplementary Information [file 41431_2021_1027_MOESM1_ESM.pdf]

Supplementary information

Complementing the phenotypical spectrum of *TUBA1A* tubulinopathy and its role in early-onset epilepsies

Schröter et al., European Journal of Human Genetics 2021

Supplementary Methods

3D protein structure modeling, VEP analyses, and literature research

Localization, structural consequence, and kinesin interaction of identified *TUBA1A* missense variants in the quaternary protein structure were analyzed and visualized as described previously using the PDB templates J5CO (TUBA1A) and 2HXF (KIF1A) (1). For visualization of variant distributions, we compiled all known pathogenic and benign *TUBA1A* missense variants as well as their respective allele count using the databases PubMed, HGMD, ClinVar, and gnomAD. PubMed research was performed using the search terms "TUBA1A" and "tubulinopathy". ClinVar variants were filtered by the categories "pathogenic", "likely pathogenic", "missense", "single nucleotide [variation]", and "clinical testing" and gnomAD variants by the category "missense only". All databases were most recently accessed 31th December 2020. Variants were standardized to the Ensembl feature ENST00000301071.7 (Ref Seq NM_006009.3) of the GRCh37/hg19 human reference genome build. Additionally, values of the established VEP scores REVEL, CADD, MetaLR, PROVEAN, PolyPhen-2, and SIFT of all biologically possible *TUBA1A* missense variants were compiled and annotated using the dbNSFP (v.4.1a) database (2-7). VEP score values and variant distributions were plotted alongside the linearized TUBA1A protein using the *ggplot2* library in RStudio (v. 1.3.1093; RStudio, Inc.). The REVEL ensemble score showed

high performance regarding discrimination between pathogenic and benign single nucleotide variants in multiple datasets (8). Furthermore, the REVEL score demonstrated statistically significant discrimination between pathogenic and benign variants in a comprehensive *TUBA1A* variant dataset (1). Therefore, REVEL score values are primarily used for variant assessment in this study.

Systematic, quantitative assessment of neuroradiological features

Nine postnatal MRIs of four subjects (i02, i03, i05, i06) and one fetal MRI (i09) were systematically reviewed for abnormalities of (1.) cerebral cortex: (a) gyral pattern, (b) thick cortex, (c) irregularity of cortical surface and/or cortex-white matter interface, (d) abnormal signal (summarized as “dysgyria”); hippocampus: (a) signal abnormalities, (b) hypoplastic (thin, incompletely enfolded), (c) as incompletely inverted (non-oval, steep and deep collateral sulcus), (d) dysplastic (thick and irregular shape), (2.) basal ganglia and thalamus, (3.) internal capsule, (4.) brainstem, (5.) posterior fossa, (6.) cerebellum, (7.) white matter including myelination and thinning/absence of the CC, (8.) cranial nerves including absence/asymmetry of olfactory bulbs and thinning/absence of the optic nerves, and (9.) ventricles and external CSF spaces. Maximum sagittal diameters of mesencephalon, pons, and medulla oblongata, anterior-posterior and cranio-caudal diameters of midline vermis, and transverse cerebellar diameter were measured and quantified as z-scores relative to postnatal, age-adapted control values (9). Regarding the fetal MRI, biparietal (BPD) and fronto-occipital diameter (FOD, both whole brain), atrial diameter (AD, ventricles), antero-posterior diameter of pons (APDP), and transverse cerebellar diameter (TCD, cerebellum) were measured and quantified as percentiles adapted to the gestational week (10, 11).

Supplementary figure and table legends

Figure S1: Individual i06 at 2.8 months (A, C) and 14.8 years (B, D), before and after shunting of the hydrocephalus. Irregular, simplified cerebral sulci with exception of bilateral focal agyria of medial parieto-occipital lobes ($A_{2,3}$). Irregular internal and external cortical surface in right temporal und bilateral perisylvian location (C_3) suggestive of polymicrogyria. Hypoplastic hippocampus without internal structure (C_4). Ballooning of temporal horns before treatment of hydrocephalus (A_2, B_2). Small thalami and dysplastic basal ganglia without discernible ALIC (A_3, $C_{3,4}$). Agenesis of the CC ($C_{1,4}$, $D_{1,4}$). Abnormal, thin brainstem with disproportionately thick mesencephalon, short pons, long medulla oblongata, and asymmetry (C_1, D_1, $A_{4,5}$, $B_{4,5}$). Hypoplastic, rotated vermis in a relatively large posterior fossa but without high insertion of the tentorium (C_1). Abnormal cerebellar foliation (A_4, C_5). Hypopolastic left olfactory bulb (C_2, D_2). Ischemia in territory of the posterior cerebral arteries including the left temporo-occipital gyri ($B_{1\text{-}3}$). Mild widening of external CSF spaces after shunting, thick skull, short sagittal skull diameter. Myelination is initially borderline without MR-visible myelin in the central region on T1w images (not shown) but complete on follow-up. Development of cerebral white matter has somewhat progressed and/or is less compressed after hydrocephalus has been treated.
Abbreviations: ALIC=anterior limb of the internal capsule; CC=corpus callosum; CSF=cerebrospinal fluid.

Figure S2: Individual i02 with caudally absent ALIC and perisylvian polymicrogyria. T2w (A_1-C_1, A_3-C_3, C_5) and T1w images (A_2-C_2, A_4-C_4, A_5, C_5) of individual 2 showing normal contour of basal ganglia and thalami compared to an age-matched individual with normal imaging ($A_{1,2}$, $B_{1,2}$). The ALIC can be distinguished from the basal ganglia by its normal signal of myelinated, T2-hypointense, T1-hyperintense white matter in its cranial

course ($A_{3,4}$), but not more caudally where it normally meets fibers from the anterior commissure ($C_{3,4}$ compared to $C_{1,2}$). Note irregularity of the inner and outer surface of insular cortex and opercula (B_4, enlarged in B_5) consistent with perisylvian polymicrogyria. CC, brainstem, and vermis were normal.

Abbreviations: CC=corpus callosum.

Figure S3: Representative EEG traces from individual i03.

(A) Continuous suppression < 10µV of brain activity with rare sub-delta waves up to 20µV at 4 days of age. During the neonatal period, polymorphic myoclonia without EEG correlates were observed. (B) During follow-up in infancy, the EEG pattern evolved to multifocal spikes with intermittent suppression of activity, resembling suppression-burst pattern. (C) At 3.5 years of age, the EEG displayed sleep-potentiated, rhythmic, generalized spike-wave activity (continuous spike-and-wave during sleep; CSWS).

Figure S4: Synopsis of heatmap models of established VEP scores for all possible missense variants according to their position in the TUBA1A primary structure.

Table S1:

Neuroradiological features of five individuals assessed by systematic, quantitative re-evaluation.

Table S2:

Variant identifiers and corresponding VEP scores of *TUBA1A* variants identified in this report.

Table S3:

All VEP scores available in the dbNFSP database of all biologically possible *TUBA1A* missense variants harmonized with the canonical Ensembl feature.

Table S4:

All *TUBA1A* variants identified in PubMed, ClinVar, and gnomAD with corresponding allele count.

Supplementary figures

Figure S1

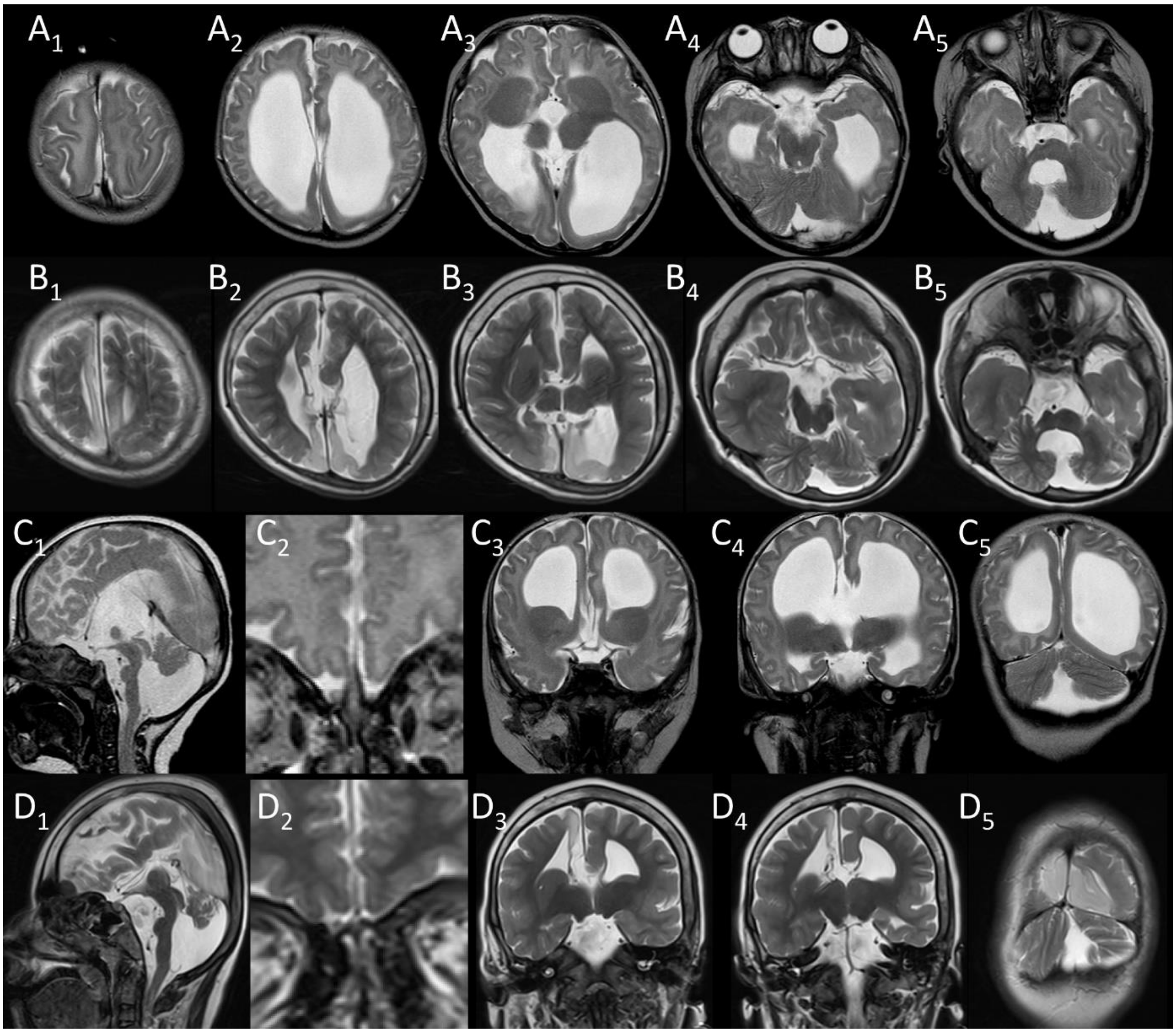

Figure S2

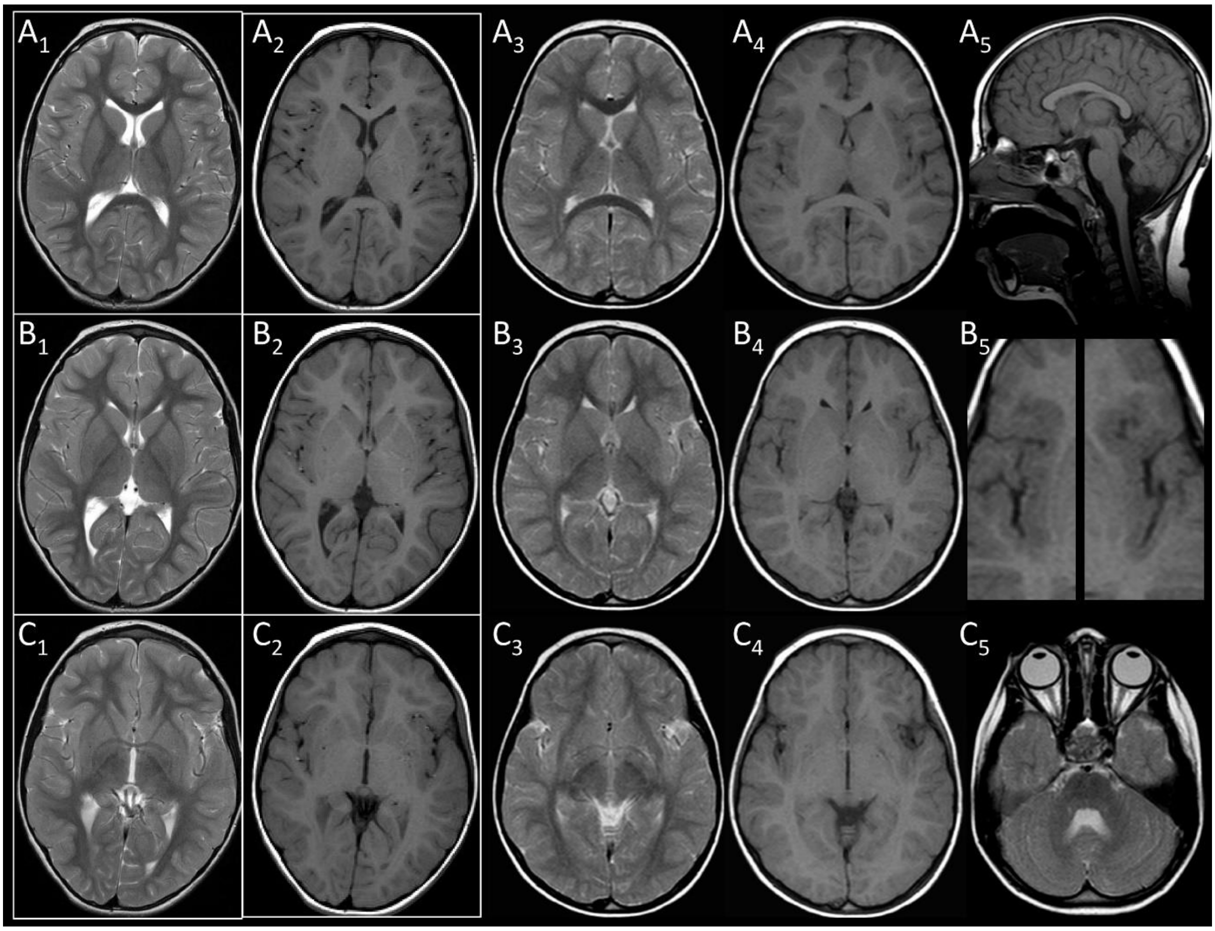

Figure S3

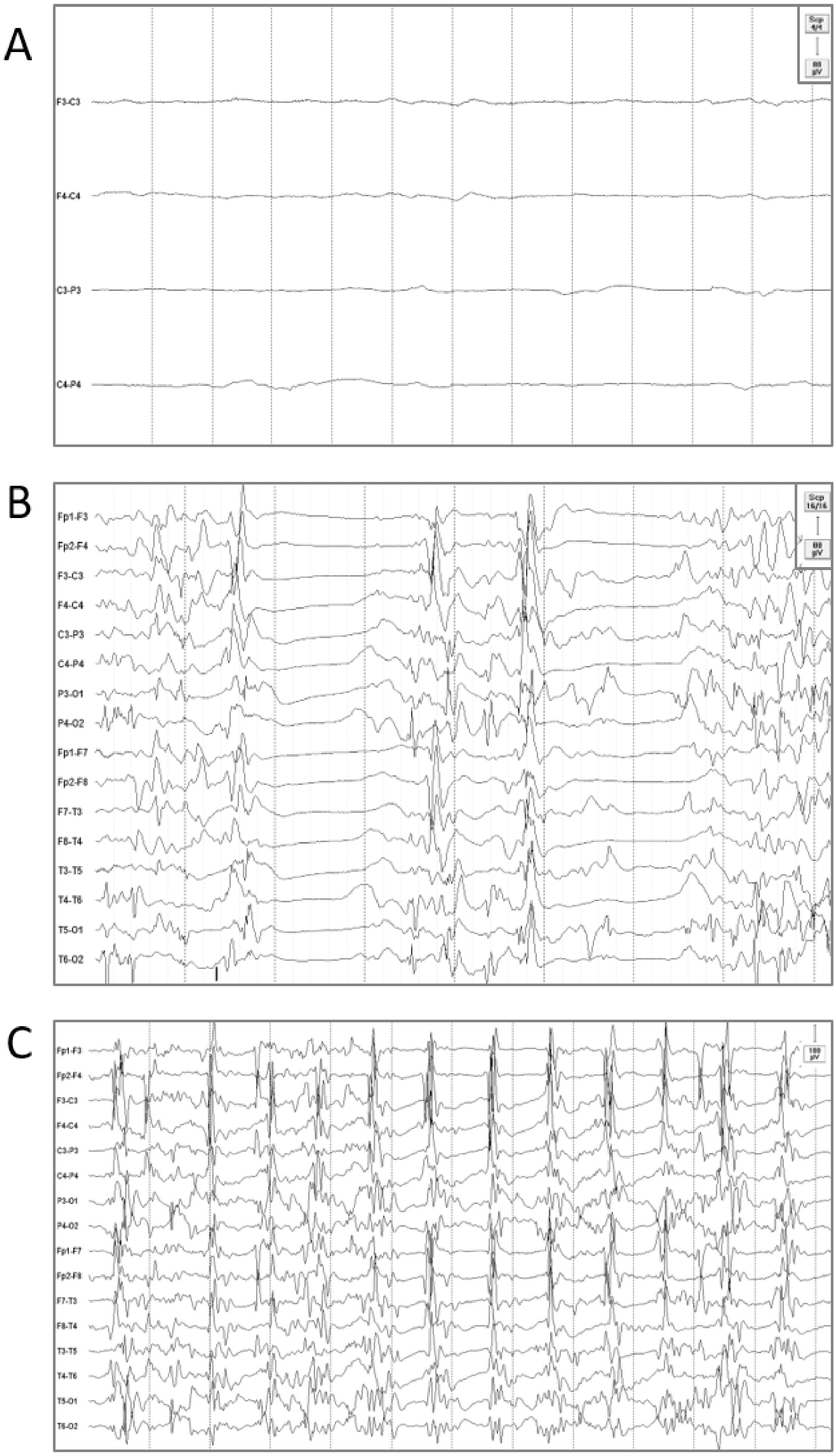
A
F3-C3
F4-C4
C3-P3
C4-P4
B
Fp1-F3
Fp2-F4
F3-C3
F4-C4
C3-P3
C4-P4
P3-O1
P4-O2
Fp1-F7
Fp2-F8
F7-T3
F8-T4
T3-T5
T4-T6
T5-O1
T6-O2
C
Fp1-F3
Fp2-F4
F3-C3
F4-C4
C3-P3
C4-P4
P3-O1
P4-O2
Fp1-F7
Fp2-F8
F7-T3
F8-T4
T3-T5
T4-T6
T5-O1
T6-O2
100 µV

Figure S4

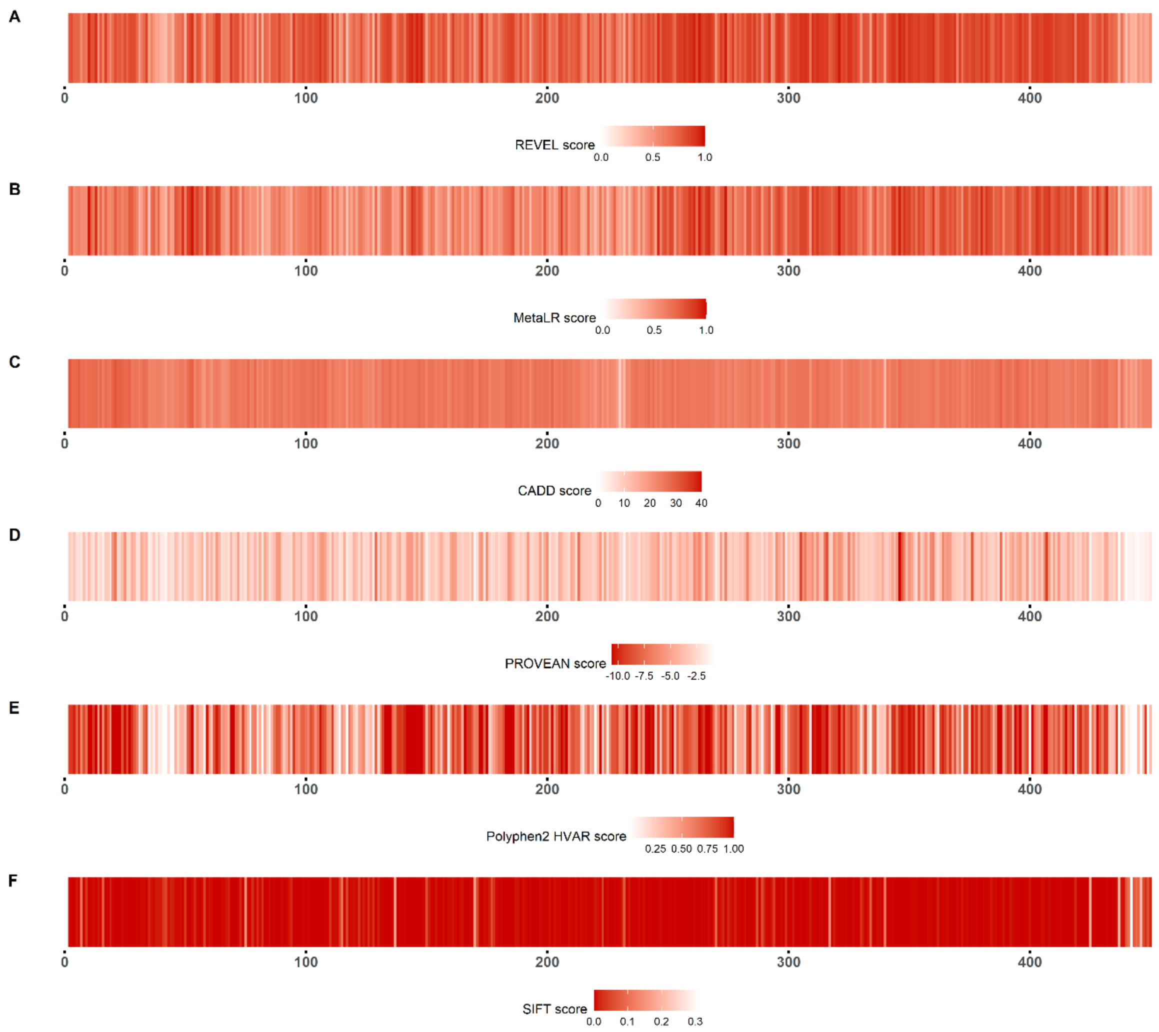
A
0
100
200
300
400
REVEL score
0.0 0.5 1.0
B
0
100
200
300
400
MetaLR score
0.0 0.5 1.0
C
0
100
200
300
400
CADD score
0 10 20 30 40
D
0
100
200
300
400
PROVEAN score
-10.0 -7.5 -5.0 -2.5
E
0
100
200
300
400
Polyphen2 HVAR score
0.25 0.50 0.75 1.00
F
0
100
200
300
400
SIFT score
0.0 0.1 0.2 0.3

Supplementary references

1. Hebebrand M, Huffmeier U, Trollmann R, Hehr U, Uebe S, Ekici AB, et al. The mutational and phenotypic spectrum of TUBA1A-associated tubulinopathy. Orphanet J Rare Dis. 2019;14(1):38.
2. Dong C, Wei P, Jian X, Gibbs R, Boerwinkle E, Wang K, et al. Comparison and integration of deleteriousness prediction methods for nonsynonymous SNVs in whole exome sequencing studies. Hum Mol Genet. 2015;24(8):2125-37.
3. Ioannidis NM, Rothstein JH, Pejaver V, Middha S, McDonnell SK, Baheti S, et al. REVEL: An Ensemble Method for Predicting the Pathogenicity of Rare Missense Variants. Am J Hum Genet. 2016;99(4):877-85.
4. Liu X, Li C, Mou C, Dong Y, Tu Y. dbNSFP v4: a comprehensive database of transcript-specific functional predictions and annotations for human nonsynonymous and splice-site SNVs. Genome Med. 2020;12(1):103.
5. Rentzsch P, Witten D, Cooper GM, Shendure J, Kircher M. CADD: predicting the deleteriousness of variants throughout the human genome. Nucleic Acids Res. 2019;47(D1):D886-D94.
6. Adzhubei IA, Schmidt S, Peshkin L, Ramensky VE, Gerasimova A, Bork P, et al. A method and server for predicting damaging missense mutations. Nat Methods. 2010;7(4):248-9.
7. Sim NL, Kumar P, Hu J, Henikoff S, Schneider G, Ng PC. SIFT web server: predicting effects of amino acid substitutions on proteins. Nucleic Acids Res. 2012;40(Web Server issue):W452-7.
8. Gunning AC, Fryer V, Fasham J, Crosby AH, Ellard S, Baple EL, et al. Assessing performance of pathogenicity predictors using clinically relevant variant datasets. J Med Genet. 2020.

9. Garbade SF, Boy N, Heringer J, Kolker S, Harting I. Age-Related Changes and Reference Values of Bicaudate Ratio and Sagittal Brainstem Diameters on MRI. Neuropediatrics. 2018;49(4):269-75.

10. Kyriakopoulou V, Vatansever D, Davidson A, Patkee P, Elkommos S, Chew A, et al. Normative biometry of the fetal brain using magnetic resonance imaging. Brain Struct Funct. 2017;222(5):2295-307.

11. Tilea B, Alberti C, Adamsbaum C, Armoogum P, Oury JF, Cabrol D, et al. Cerebral biometry in fetal magnetic resonance imaging: new reference data. Ultrasound Obstet Gynecol. 2009;33(2):173-81.
